# Development and Validation of the Elecsys Anti-SARS-CoV-2 Immunoassay as a Highly Specific Tool for Determining Past Exposure to SARS-CoV-2

**DOI:** 10.1128/JCM.01694-20

**Published:** 2020-09-22

**Authors:** Peter Muench, Simon Jochum, Verena Wenderoth, Beatus Ofenloch-Haehnle, Michael Hombach, Matthias Strobl, Henrik Sadlowski, Christopher Sachse, Giulia Torriani, Isabella Eckerle, Alexander Riedel

**Affiliations:** a Roche Diagnostics GmbH, Penzberg, Germany; b Roche Diagnostics International Ltd, Rotkreuz, Switzerland; c Labor Berlin–Charité Vivantes Services GmbH, Berlin, Germany; d KRH Labor GmbH, Hannover, Germany; e Department of Molecular Medicine and Microbiology, Faculty of Medicine, Université de Genève, Geneva, Switzerland; f Hôpitaux Universitaires Genève, Geneva, Switzerland; g Division of Infectious Diseases, Department of Medicine, Université de Genève, Geneva, Switzerland; h Laboratory of Virology, Division of Laboratory Medicine, Université de Genève, Geneva, Switzerland; i Center for Emerging Viral Diseases, Université de Genève, Geneva, Switzerland; Cepheid

**Keywords:** SARS-CoV-2, cross-reactivity, diagnosis, immunoassay, neutralization testing, sensitivity, specificity

## Abstract

The Elecsys Anti-SARS-CoV-2 immunoassay (Roche Diagnostics) was developed to provide accurate, reliable detection of antibodies to severe acute respiratory syndrome coronavirus 2 (SARS-CoV-2). We evaluated sensitivity, specificity, cross-reactivity, and agreement with a vesicular stomatitis virus-based pseudoneutralization assay for the Elecsys Anti-SARS-CoV-2 immunoassay. Sensitivity and agreement between Elecsys Anti-SARS-CoV-2 immunoassay and pseudoneutralization assay measurements were evaluated using samples from patients with PCR-confirmed SARS-CoV-2 infection, a majority of whom were hospitalized. Specificity was evaluated using samples from routine diagnostic testing/blood donors collected before December 2019 and thus deemed negative for SARS-CoV-2-specific antibodies. Cross-reactivity was evaluated using samples containing a wide range of potentially cross-reacting analytes, purchased from commercial vendors. For sensitivity and specificity, point estimates and 95% confidence intervals (CIs) were calculated. Agreement between the Elecsys Anti-SARS-CoV-2 immunoassay and the pseudoneutralization assay was calculated. The sensitivity of the Elecsys Anti-SARS-CoV-2 immunoassay in patients with prior PCR-confirmed SARS-CoV-2 infection was 99.5% (95% CI, 97.0 to 100.0%) at ≥14 days post-PCR confirmation. Overall specificity (*n* = 10,453) was 99.80% (95% CI, 99.69 to 99.88%). Only 4/792 samples containing potential cross-reacting analytes were reactive with the Elecsys Anti-SARS-CoV-2 immunoassay, resulting in an overall specificity in this cohort of 99.5% (95% CI, 98.6 to 99.9%). Positive, negative, and overall agreement (*n* = 46) between the Elecsys Anti-SARS-CoV-2 immunoassay and the pseudoneutralization assay were 86.4% (95% CI, 73.3 to 93.6%), 100% (95% CI, 34.2 to 100%), and 87.0% (95% CI, 74.3 to 93.9%), respectively. The Elecsys Anti-SARS-CoV-2 immunoassay demonstrated high sensitivity (99.5% at ≥14 days post-PCR confirmation) and specificity (99.80%), supporting its use as a tool for identification of past SARS-CoV-2 infection, including use in populations with low disease prevalence.

## Abstract (precis)

The Elecsys Anti-SARS-CoV-2 immunoassay (Roche Diagnostics) was developed to provide accurate, reliable detection of antibodies to severe acute respiratory syndrome coronavirus 2 (SARS-CoV-2). We evaluated sensitivity, specificity, cross-reactivity, and agreement with a vesicular stomatitis virus-based pseudoneutralization assay for the Elecsys Anti-SARS-CoV-2 immunoassay. Sensitivity and agreement between Elecsys Anti-SARS-CoV-2 immunoassay and pseudoneutralization assay measurements were evaluated using samples from patients with PCR-confirmed SARS-CoV-2 infection, a majority of whom were hospitalized.

## INTRODUCTION

In December 2019, reports emerged of patients presenting with pneumonia of unknown etiology in Wuhan, China (1, 2). This disease was subsequently shown to be caused by a novel coronavirus, severe acute respiratory syndrome coronavirus 2 (SARS-CoV-2), and was designated coronavirus disease 2019 (COVID-19) (3–5). Since then, the COVID-19 outbreak has rapidly developed into a pandemic, which has infected millions of people and posed critical challenges for governments and health care systems around the world (6).

SARS-CoV-2 is an enveloped, single-stranded RNA virus of the *Coronaviridae* family. All coronaviruses share similarities in the organization and expression of their genomes, which encode 16 nonstructural proteins and 4 structural proteins: the spike, envelope, membrane, and nucleocapsid antigens (5, 7–9). Evidence to date suggests that SARS-CoV-2 is transmitted between people primarily through respiratory droplets and contact routes, although indirect transmission via contaminated surfaces is also possible (10–12). Infected individuals may exhibit a variety of symptoms, including fever, cough, and breathlessness, and disease severity can range from asymptomatic/mild cases to severe disease and death (13, 14).

There is an urgent unmet clinical need to more effectively determine SARS-CoV-2 seroprevalence in the general population in order to improve our understanding of virus circulation dynamics, gain a more accurate estimate of the mortality rate from COVID-19, and identify individuals at risk of infection. Serological assays for SARS-CoV-2 have been suggested as a potential tool to help identify the extent of virus exposure in a given population and thereby indirectly provide information on the appropriate application, enforcement, or relaxation of containment measures (15–18). Serological assays may also help elucidate a potential correlate for immunity following infection (15, 16).

Recent evidence suggests that most SARS-CoV-2 convalescent individuals have detectable neutralizing antibodies (nAbs) for the virus (19, 20). Due to affinity maturation, the binding strength of antibodies increases over time following infection or vaccination (21). High-affinity nAbs are critical for the control of infection, since they can recognize and bind to specific viral epitopes, thereby “neutralizing” the virus and rendering it nonpathogenic (20, 22). Previous studies involving commercially available anti-SARS-CoV-2 immunoassays have found a positive correlation between antibody titration results from pseudoneutralization assays and SARS-CoV-2 nAbs; however, further investigation is warranted (23, 24).

The timing of seroconversion is crucial for determining optimum time points for sample collection for serological testing (25). Although the picture is rapidly developing and robust serology data are not yet available, the kinetics of antibodies to SARS-CoV-2 have begun to be described. Based on current evidence, immunoglobulin M (IgM) antibodies are detectable within 5 days after symptom onset and immunoglobulin G (IgG) antibodies within 5 to 7 days (26–28). There is a paucity of data on immunoglobulin A (IgA), but it appears to be observable approximately 3 to 6 days after symptom onset (15, 27). Depending on the method applied, seroconversion is observed after a median of 10 to 13 days after symptom onset for IgM and 12 to 14 days for IgG; maximum seroconversion occurs at 2 to 3 weeks for IgM, 3 to 6 weeks for IgG, and 2 weeks for total antibodies (20, 28–32). The levels and chronological order of IgM and IgG antibody appearance are highly variable, supporting the detection of both antibodies simultaneously (17, 28, 29, 31).

The Elecsys Anti-SARS-CoV-2 immunoassay (Roche Diagnostics International Ltd, Rotkreuz, Switzerland) was developed to provide an accurate and reliable method for the detection of antibodies to SARS-CoV-2, in order to facilitate population screening with high specificity and the identification of past infection status as a potential correlate for subsequent immunity. We aimed to evaluate the sensitivity, specificity, and cross-reactivity of the Elecsys Anti-SARS-CoV-2 immunoassay, in addition to agreement with results from a pseudoneutralization assay.

## MATERIALS AND METHODS

### Study design.

The performance of the Elecsys Anti-SARS-CoV-2 immunoassay was prospectively evaluated at Roche Diagnostics (Penzberg, Germany). Sensitivity and specificity analyses were conducted using anonymized residual frozen samples from routine diagnostic testing or from blood donors, which were obtained from diagnostic laboratories in Germany (Labor Berlin–Charité Vivantes Services GmbH, Berlin, Germany, and KRH Labor GmbH, Hannover, Germany) and a blood product provider in the United States (Golden West Biologicals, CA, USA), respectively. All samples utilized for sensitivity analyses were from patients with PCR-confirmed SARS-CoV-2 infection. Cross-reactivity analyses were conducted using anonymized frozen samples containing potentially cross-reacting factors, which were purchased from various commercial vendors. For analysis of agreement between the Elecsys Anti-SARS-CoV-2 immunoassay and a vesicular stomatitis virus (VSV)-based pseudoneutralization assay, anonymized residual frozen samples from patients with PCR-confirmed SARS-CoV-2 infection were used.

The study was conducted in accordance with applicable regulations, including relevant European Union directives and regulations, and the principles of the Declaration of Helsinki. All samples, excluding the specimens that were provided by commercial sample vendors, were transferred to Roche following anonymization. For studies with anonymized leftover specimens, no ethics committee vote is required. A statement was obtained from the Ethics Committee of the Landesärztekammer Bayern confirming that there are no objections to the transfer and coherent use of anonymized leftover samples.

### Assay.

The Elecsys Anti-SARS-CoV-2 electrochemiluminescence immunoassay is intended for use on cobas e analyzers (Roche Diagnostics International Ltd, Rotkreuz, Switzerland) for *in vitro* qualitative detection of antibodies (including both IgA and IgG) to SARS-CoV-2 in human serum and plasma. The immunoassay utilizes a double-antigen sandwich test principle and a recombinant protein representing the nucleocapsid antigen for the determination of antibodies to SARS-CoV-2.

For the present study, the Elecsys Anti-SARS-CoV-2 immunoassay was performed according to the manufacturer’s instructions, and assay results were interpreted as follows: cutoff index, <1.0 for samples that were nonreactive/negative for anti-SARS-CoV-2 antibodies; cutoff index, ≥1.0 for samples that were reactive/positive for anti-SARS-CoV-2 antibodies.

### Sensitivity.

The sensitivity of the Elecsys Anti-SARS-CoV-2 immunoassay was evaluated using residual samples from patients who had previously tested positive for SARS-CoV-2 infection by PCR. One or more consecutive samples were collected from patients at various time points after PCR confirmation. All samples taken from the same donor on the same day were excluded, leaving only multiple donations from one donor if taken on different calendar days. Samples derived from all patients with prior PCR-confirmed SARS-CoV-2 infection were used; no additional sample selection was made except for the availability of a sufficient amount of residual serum or plasma. Assay sensitivity at different time points after PCR confirmation was calculated as the percentage of samples that tested positive with the Elecsys Anti-SARS-CoV-2 immunoassay relative to the total number of PCR-confirmed positive samples.

### Specificity.

The specificity of the Elecsys Anti-SARS-CoV-2 immunoassay was evaluated using unselected residual samples from routine diagnostic testing or from blood donors. All samples were collected before December 2019 (i.e., before the first description of an infection with SARS-CoV-2) and were thus deemed negative for SARS-CoV-2-specific antibodies. Assay specificity was calculated as the percentage of true SARS-CoV-2 antibody-negative samples that tested negative with the Elecsys Anti-SARS-CoV-2 immunoassay.

### Cross-reactivity.

The cross-reactivity of the Elecsys Anti-SARS-CoV-2 immunoassay was evaluated using samples collected before December 2019 that had previously been characterized as positive for a wide range of different indications, including common cold (donors selected by symptoms only) and coronavirus (229E, NL63, OC43, and HKU1) panels, and samples from patients with autoimmune conditions and other diseases associated with a higher prevalence of autoantibodies and immune dysfunction, which may increase the risk of interference with serological testing. Assay specificity was calculated for each potential cross-reactive sample type and for the total cohort as the percentage of samples tested that were reactive with the Elecsys Anti-SARS-CoV-2 immunoassay.

### Agreement between the Elecsys Anti-SARS-CoV-2 immunoassay and neutralization testing (VSV-based pseudoneutralization assay).

The diagnostic accuracy of the Elecsys Anti-SARS-CoV-2 immunoassay was compared with that of a VSV-based pseudoneutralization assay at an external laboratory in Geneva, Switzerland (Hôpitaux Universitaires Genève), using residual frozen samples from patients with PCR-confirmed SARS-CoV-2 infection. A titer of 1:20 (corresponding to the titer at which 50% pseudoneutralization activity was observed) was used as the positive cutoff for the pseudoneutralization assay. Discrepant samples were retested using an in-house SARS-CoV-2 whole spike protein-based immunofluorescence assay and were excluded if indeterminate results were obtained. Full details of the methodology used for VSV-based pseudoneutralization assay measurements have been published previously by Meyer et al. (33). The percent positive agreement (PPA), percent negative agreement (PNA), and percent overall agreement (POA) rates were determined, and 95% confidence intervals (CIs) were calculated.

### Statistical analyses.

Sample size estimation for the specificity analyses was calculated using the binomial exact test (34). The following sample sizes for a seronegative cohort would be required to obtain an alpha value of 5% and a power of 80%: null proportion (*p*0) = 0.99, *n *= 368; *p*0 = 0.995, *n *= 736; *p*0 = 0.996, *n *= 921; *p*0 = 0.997, *n *= 1,228; *p*0 = 0.998, *n *= 1,843; *p*0 = 0.999, *n *= 3,688; *p*0 = 0.9995, *n *= 7,376. A sample size (*n*) of >10,000 would ensure that the analyses were sufficiently powered to provide a reliable estimate of assay specificity. For the cross-reactivity analyses, the aim was to evaluate ≥10 samples per cross-reacting factor.

Determinations were performed as single measurements. For sensitivity and specificity, point estimates and 95% CIs were calculated using the Roche Diagnostics bioWARP tool (Roche Diagnostics GmbH, Penzberg, Germany). For contingency between Elecsys Anti-SARS-CoV-2 immunoassay and VSV-based pseudoneutralization assay results, PPA, PNA, and POA were calculated using the Westgard QC 2×2 Contingency Calculator (Westgard QC Inc., WI, USA).

### Data availability.

Qualified researchers may request access to individual-patient-level data through the clinical study data request platform (https://vivli.org/). Details on Roche's criteria for eligible studies are available at https://vivli.org/members/ourmembers/. Further details on Roche's Global Policy on the Sharing of Clinical Information and how to request access to related clinical study documents are available on the company website.

## RESULTS

### Sensitivity.

**FIG 1:**
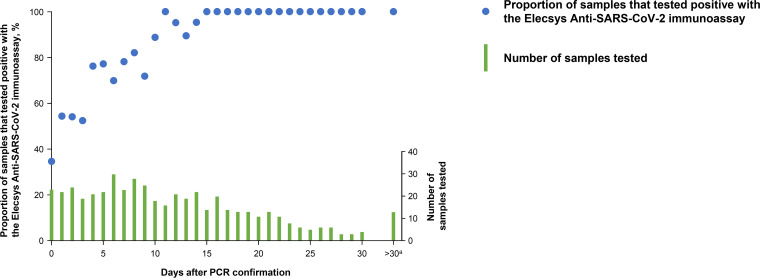
Sensitivity of the Elecsys Anti-SARS-CoV-2 immunoassay in patients with prior PCR-confirmed SARS-CoV-2 infection. ^a^The maximum period after a positive PCR result was 44 days (one patient).

A total of 496 samples from 102 patients with prior PCR-confirmed SARS-CoV-2 infection were included in the sensitivity analyses. All positive-sample donors (anonymized and aggregated data) were adults (mean [standard deviation {SD}] age, 66 [16.4] years) and had an average length of stay in hospital of 18 (SD, 14.9) days. Overall, 15.7% of the sample donors were admitted to an intensive care unit during the hospital stay. Sensitivity increased with time after PCR confirmation: 60.2% (95% CI, 52.3 to 67.8%) at 0 to 6 days, 85.3% (95% CI, 78.6 to 90.6%) at 7 to 13 days, and 99.5% (95% CI, 97.0 to 100.0%) at ≥14 days (Table 1; Fig. 1).

**TABLE 1:** Summary of sensitivity results for the Elecsys Anti-SARS-CoV-2 immunoassay in patients with prior PCR-confirmed SARS-CoV-2 infection

Days after PCR confirmation	No. of donors^*a*^	No. of samples tested			Sensitivity (% [95% CI])
		Total	Reactive	Nonreactive	
0–6	75	161	97	64	60.2 (52.3–67.8)
7–13	52	150	128	22	85.3 (78.6–90.6)
≥14	41	185	184	1^*b*^	99.5 (97.0–100.0)

a The total number of donors was 102. Some donors did not provide samples for all three time frames.

b One patient was nonreactive at day 14 (cutoff index, 0.7) but reactive at day 16 (cutoff index, 4.5).

**FIG 2:**
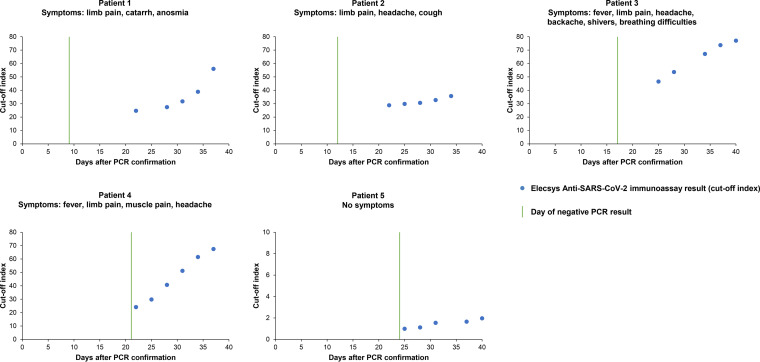
Elecsys Anti-SARS-CoV-2 immunoassay results for 26 consecutive samples from five patients following recovery from PCR-confirmed SARS-CoV-2 infection. Day 0 represents the day of the initial positive PCR result.

Figure 2 shows Elecsys Anti-SARS-CoV-2 immunoassay results for 26 consecutive samples from five patients following recovery from PCR-confirmed SARS-CoV-2 infection. Patients had a range of SARS-CoV-2 symptoms, including one patient who reported no symptoms. In all patients with symptoms, cutoff index values were ≥20 following a negative PCR result and increased over time, out to day 40. In the patient with no symptoms, cutoff index values ranged from 0.99 to 1.97 following a negative PCR result.

### Specificity.

A total of 10,453 samples were included in the specificity analyses: 6,305 samples from routine diagnostic testing and 4,148 samples from blood donors. The overall specificity for the entire sample cohort was 99.80% (95% CI, 99.69 to 99.88%); specificity was 99.81% (95% CI, 99.67 to 99.90%) in routine diagnostic samples and 99.78% (95% CI, 99.59 to 99.90%) in blood donor samples (Table 2).

**TABLE 2:** Summary of specificity results for the Elecsys Anti-SARS-CoV-2 immunoassay in residual samples from routine diagnostic testing and blood donors

Sample cohort	No. of samples tested			Specificity (% [95% CI])
	Total	Reactive	Nonreactive	
Routine diagnostic samples	6,305	12	6,293	99.81 (99.67–99.90)
Blood donor samples	4,148	9	4,139	99.78 (99.59–99.90)
Total	10,453	21	10,432	99.80 (99.69–99.88)

### Cross-reactivity.

Out of 792 samples with potential cross-reactivity, 4 samples were reactive with the Elecsys Anti-SARS-CoV-2 immunoassay: 1/85 (1.2%) acute cytomegalovirus infection samples, 2/105 (1.9%) acute Epstein-Barr virus infection samples, and 1/10 (10.0%) systemic lupus erythematosus samples (Table 3). Of the samples that were tested for potential cross-reactivity with common cold (*n *= 40) and coronavirus (*n *= 40) (229E, NL63, OC43, HKU1) panels, none were reactive. The overall specificity in this cohort was 99.5% (95% CI, 98.6 to 99.9%) (Table 3).

**TABLE 3:** Summary of cross-reactivity results for the Elecsys Anti-SARS-CoV-2 immunoassay

Potential cross-reactive sample type^*a*^	Vendor	No. of samples tested		Specificity (%)
		Total	Reactive	
Common cold panel	Roche Diagnostics (internal)	40	0	100.0
Coronavirus panel^*b*^	Academic collaboration (noncommercial)	40	0	100.0
Cytomegalovirus, acute infection (IgM and IgG positive)	German Red Cross	85	1	98.8
Epstein-Barr virus, acute infection (EBV IgM and EBV VCA IgG positive)	Cerba HealthCare and AML Diagnostics	105	2	98.1
*Borrelia* (IgM positive)	Roche Diagnostics (internal)	6	0	100.0
Chlamydia pneumoniae (IgG positive)	Cerba HealthCare	8	0	100.0
Escherichia coli (anti-E. coli reactive)	Trina Bioreactives	10	0	100.0
Gonorrhoea (symptomatic, Gram stain positive)	BBI Diagnostics and ZeptoMetrix	5	0	100.0
Hepatitis A virus, acute infection (IgM positive)	Trina Bioreactives	10	0	100.0
Hepatitis A virus, late infection (IgG positive)	Roche Diagnostics (internal)	15	0	100.0
Hepatitis A virus vaccinees (anti-HAV total positive and anti-HAV IgM negative)	Roche Diagnostics (internal)	15	0	100.0
Hepatitis B virus, early acute infection (HBsAg/HBeAg positive)	Trina Bioreactives	12	0	100.0
Hepatitis B virus, acute infection (anti-HBs positive)	Trina Bioreactives	7	0	100.0
Hepatitis B virus, acute infection (anti-HBc IgM positive)	Trina Bioreactives	8	0	100.0
Hepatitis B virus, chronic infection (HBsAg reactive, HBeAg negative)	Trina Bioreactives	12	0	100.0
Hepatitis B virus vaccinees (confirmed vaccination)	Roche Diagnostics (internal)	15	0	100.0
Hepatitis C virus, acute infection (IgM positive)	Trina Bioreactives	6	0	100.0
Hepatitis C virus (IgG positive)	Trina Bioreactives	60	0	100.0
Hepatitis E virus (IgG positive)	Biomex	12	0	100.0
Human immunodeficiency virus (anti-HIV and/or HIV Ag reactive)	Academic collaboration (noncommercial) and home-based tests	10	0	100.0
Herpes simplex virus, acute infection (IgM positive)	Trina Bioreactives	24	0	100.0
Human T-lymphotropic virus (anti-HTLV total reactive)	U.S. Red Cross	6	0	100.0
Influenza vaccinees (confirmed vaccination)	Roche Diagnostics (internal)	25	0	100.0
Listeria (antibody positive)	Cerba HealthCare	6	0	100.0
Measles (IgM and IgG positive)	ZeptoMetrix	10	0	100.0
Mumps (IgM and IgG positive)	ZeptoMetrix	14	0	100.0
Parvovirus B19 (IgM and IgG positive)	Cerba HealthCare	30	0	100.0
Plasmodium falciparum (malaria; IF positive)	Trina Bioreactives	8	0	100.0
Rubella, acute infection (IgM and IgG positive)	Biomex	12	0	100.0
Toxoplasma gondii (IgM and IgG positive)	DiaServe Laboratories GmbH and Trina Bioreactives	8	0	100.0
Treponema pallidum (syphilis; anti-syphilis total positive)	IMPATH-BCP, BBI Diagnostics, and ZeptoMetrix	62	0	100.0
Varicella-zoster virus (IgG positive)	BBI Diagnostics	30	0	100.0
Anti-mitochondrial antibodies	Trina Bioreactives	30	0	100.0
Anti-nuclear antibodies (IF positive)	Cerba HealthCare	26	0	100.0
Systemic lupus erythematosus	iSpecimen	10	1	90.0
Rheumatoid arthritis	iSpecimen	10	0	100.0
Total		792	4	99.5 (95% CI, 98.6–99.9)

a Anti-HBc, antibodies to hepatitis B core antigen; anti-HBs, antibodies to hepatitis B surface antigen; EBV, Epstein-Barr virus; HBeAg, hepatitis B envelope antigen; HBsAg, hepatitis B surface antigen; HIV, human immunodeficiency virus; HTLV, human T-lymphotropic virus; IgG, immunoglobulin G; IgM, immunoglobulin M; VCA, viral capsid antigen.

b Samples from individuals following a PCR-confirmed infection with human coronavirus 229E, NL63, OC43, or HKU1.

### Agreement between the Elecsys Anti-SARS-CoV-2 immunoassay and neutralization testing (VSV-based pseudoneutralization assay).

In total, 47 samples were evaluated using the Elecsys Anti-SARS-CoV-2 immunoassay and a VSV-based pseudoneutralization assay for the purpose of method comparison. One sample was positive on the Elecsys Anti-SARS-CoV-2 immunoassay but indeterminate using both the VSV-based pseudoneutralization assay and in-house immunofluorescence assays, so it was excluded from the analysis. Of the remaining 46 samples, 38 tested positive on both the Elecsys Anti-SARS-CoV-2 immunoassay and the VSV-based pseudoneutralization assay (Table 4). Two samples were negative on both assays, with very low cutoff indexes observed for measurements taken using the Elecsys Anti-SARS-CoV-2 immunoassay (0.067 and 0.082). Six samples that tested positive using the VSV pseudoneutralization assay were negative on the Elecsys Anti-SARS-CoV-2 immunoassay; these samples had elevated cutoff indexes (0.206 to 0.659) compared with the two samples confirmed negative on both assays. When retested using an in-house immunofluorescence assay, two of these samples were found to be positive and four negative; all results were close to prespecified cutoffs.

**TABLE 4:** Comparison of results from the Elecsys Anti-SARS-CoV-2 immunoassay and a VSV-based pseudoneutralization assay in residual frozen samples from patients with PCR-confirmed SARS-CoV-2 infection

Result by the Elecsys Anti-SARS-CoV-2 immunoassay	Result by the VSV-based pseudoneutralization assay		
	Positive	Negative	Total
Positive	38	0	38
Negative	6	2	8
Total	44	2	46

The PPA between the Elecsys Anti-SARS-CoV-2 immunoassay and the VSV pseudoneutralization assay was 86.4% (95% CI, 73.3 to 93.6%); the PNA was 100% (95% CI, 34.2 to 100%); and the POA was 87.0% (95% CI, 74.3 to 93.9%).

## DISCUSSION

The COVID-19 pandemic has resulted in significant mortality and morbidity and has created major challenges for governments and health care systems. Due to the novelty of the causative agent, SARS-CoV-2, and the rapidity with which it has spread, there is an urgent need for serological assays, which could be used to determine the seroprevalence in a given population and help identify a potential correlate for immunity secondary to exposure (15–18). This urgency and the lack of existing available tests prompted the U.S. Food and Drug Administration to allow manufacturers of serological assays for SARS-CoV-2 to bypass the normal approval process; as a result, the market has been inundated with tests, many of which have not been sufficiently reviewed by regulatory authorities nor their performance reported in the peer-reviewed literature (16). However, it is vital that any new test be adequately evaluated and validated to demonstrate that it is reliable and accurate for its intended purpose.

To enable population screening to determine the level of exposure and to identify individuals who may be immune following infection, an ideal serological test for SARS-CoV-2 should meet several key requirements: (i) a high general specificity with a small CI, (ii) no cross-reactivity with other endemic coronaviruses, (iii) preference for the detection of mature antibodies to provide the potential for correlation with neutralizing activity, and (iv) a high sample throughput to meet the huge demand for testing. The first three requirements can be achieved through the application of an appropriate assay format and antigen selection, while the last requirement relies on a high-throughput platform and upscaling of laboratories. Although a high assay sensitivity is desirable, it is of secondary importance and should not be pursued at the expense of specificity for past infection, since a serological assay is less likely to be used for the diagnosis of active infection.

The Elecsys Anti-SARS-CoV-2 immunoassay was specifically designed to meet these requirements. It utilizes a double-antigen sandwich test principle for the detection of high-affinity (i.e., late-onset/mature) antibodies to SARS-CoV-2. In the present study, the Elecsys Anti-SARS-CoV-2 immunoassay demonstrated an overall specificity of 99.80% (95% CI, 99.69 to 99.88%) in 10,453 residual samples from routine testing and blood donors. The sensitivity of the Elecsys Anti-SARS-CoV-2 immunoassay in samples from patients with prior PCR-confirmed SARS-CoV-2 infection increased with time after PCR confirmation, reaching 99.5% (95% CI, 97.0 to 100.0%) at ≥14 days. The performance of the Elecsys Anti-SARS-CoV-2 immunoassay is comparable to or better than that observed for other serological SARS-CoV-2 assays (specificity, 94.8 to 99.9%; sensitivity at ≥14 days post-PCR confirmation, 75.0 to 100.0%) (35, 36).

The main risk with the use of serological SARS-CoV-2 assays for population screening is the possibility of false-positive results, which could lead to the erroneous assumption of past infection and subsequent putative immunity and thus could put the individual at risk of acquiring or transmitting infection (16). A very high specificity is crucial to reduce the rate of false-positive results, particularly in populations with low seroprevalence, where small differences in assay specificity can result in substantial differences in the positive predictive value (PPV) (15, 16). For example, if the seroprevalence of SARS-CoV-2 in a given population was 10%, a serological test with a sensitivity of 83.1% and a specificity of 98.3% would have a PPV of 84.2%; however, if the seroprevalence was 1%, the respective PPV would drop to 32.6% (37). Understanding this relationship between seroprevalence, specificity, and PPV is crucial for population screening using serological testing, and it has been suggested that assay specificity should be >99% (with a CI of 99.0 to 99.9%) to ensure a sufficient PPV in populations with low seroprevalence (16). Based on a sensitivity of 99.5% and a specificity of 99.8%, the PPV of the Elecsys Anti-SARS-CoV-2 immunoassay in populations of 1%, 5%, 10%, and 20% seroprevalence would be 83.4%, 96.3%, 98.2%, and 99.2%, respectively. A potential cause of false-positive results is cross-reactivity with other analytes. In the present study, only 4/792 samples containing potential cross-reacting analytes showed reactivity with the Elecsys Anti-SARS-CoV-2 immunoassay; importantly, no cross-reactivity was observed for the coronavirus panel (containing strains 229E, NL63, OC43, and HKU1). This resulted in an overall specificity of 99.5%.

The present findings indicate that a positive test result from the Elecsys Anti-SARS-CoV-2 immunoassay correlates well with *in vitro* neutralization activity; good PPA (86.4%), PNA (100%), and POA (87.0%) were observed relative to results from a VSV-based pseudoneutralization assay. However, it should be noted that the PNA reported herein is based on only two isolates and thus requires further validation. These findings are in overall agreement with those of Müller et al., who compared four commercially available serological assays for SARS-CoV-2 (the Elecsys Anti-SARS-CoV-2 immunoassay, EuroImmun anti-SARS-CoV-2 IgA and IgG enzyme-linked immunosorbent assay [ELISA], Abbott SARS-CoV-2 IgG chemiluminescent microparticle immunoassay [CMIA], and Liaison SARS-CoV-2 S1/S2 IgG chemiluminescent immunoassay [CLIA]) with a neutralization assay and observed good PPA (65.4%), PNA (100%), and POA (78.6%) for the Elecsys Anti-SARS-CoV-2 immunoassay (23). Our results also align with those of Kohmer et al., who compared six commercially available serological assays for SARS-CoV-2 (the Elecsys Anti-SARS-CoV-2 immunoassay, EuroImmun anti-SARS-CoV-2 IgG ELISA, Abbott SARS-CoV-2 IgG CMIA, Liaison SARS-CoV-2 S1/S2 IgG CLIA, VirClia COVID-19 IgG Monotest CLIA, and Virotech SARS-CoV-2 IgG ELISA) with an in-house plaque reduction neutralization test and observed good PPA (75.6%), PNA (97.1%), and POA (84.8%) (38). Nucleocapsid- and spike-based assays showed equal agreement with neutralization assays in each of these studies; however, it should be noted that the VSV-based pseudoneutralization assay utilized herein expresses only the SARS-CoV-2 spike protein and is therefore capable of capturing only spike-targeted antibodies. This should be taken into consideration when one is comparing nucleocapsid-based serological immunoassays, such as the Elecsys Anti-SARS-CoV-2 immunoassay, with a pseudoneutralization assay.

Based on our understanding of other respiratory viruses, it has been suggested that the presence of anti-SARS-CoV-2 antibodies will provide some immunity, although it is unclear how long any protection would last after the initial infection (39). Assuming that anti-SARS-CoV-2 antibodies do offer some immunity from further infection, donated plasma from patients who have recovered from COVID-19 may represent a potential therapy by conferring immunity on the recipient (39). In this context, serological assays could play another important role in the identification of potential convalescent plasma donors, particularly individuals with very high anti-SARS-CoV-2 antibody titers (16). Recent studies suggest that both cell-mediated and humoral immune responses are likely to play a protective role in SARS-CoV-2 infection, and the spike and nucleocapsid antigens in particular have been shown to be highly immunogenic and abundantly expressed during infection (7–9, 40). Antibodies targeting these proteins are formed as early as 9 days after symptom onset and have demonstrated a strong neutralizing response, suggesting that seroconversion may lead to immunity for a limited time after infection (15, 17, 41). However, further research is needed to demonstrate the correlation between the presence of anti-SARS-CoV-2 antibodies and neutralization, as well as, ultimately, the correlation with clinical immunity. Additional work is also required to establish whether there is a correlation between anti-SARS-CoV-2 antibody titers and disease severity and/or prognosis (42).

A major strength of this study is the large number of seronegative samples (*n *= 10,453) used to determine the specificity of the Elecsys Anti-SARS-CoV-2 immunoassay, which ensures that the study is robustly powered and the reliability of the specificity point estimates is very high (as indicated by the small CIs) (16, 43). To our knowledge, this is one of the largest sample sizes used to date to evaluate the specificity of a serological assay for SARS-CoV-2. Another study strength is the inclusion of a high number of confounder samples in the cross-reactivity cohort, which comprised a total of 792 samples across 36 different indications. A limitation is that this was a single-center study, and our results should be confirmed by additional assessments at other study sites. Further clinical data on the samples were not available due to data regulations, and thus, it was not possible to analyze specific subcohorts according to age, disease severity, onset of symptoms, etc. In addition, a comparably small sample size was utilized to measure agreement between the Elecsys Anti-SARS-CoV-2 immunoassay and a pseudoneutralization assay; as such, these data would benefit from further evaluation and validation. We are confident that our results regarding the sensitivity of the Elecsys Anti-SARS-CoV-2 immunoassay and the time course of antibody response are of general value. However, the findings should be interpreted with some caution. Although the samples used in this study were not selected on the basis of patient criteria, the majority of samples were drawn from hospitalized patients and therefore probably represent more-severe cases of COVID-19. Furthermore, the availability of samples depended on the need for consequent routine clinical chemistry diagnosis after PCR confirmation of SARS-CoV-2 infection. Again, it can be assumed that extensive subsequent diagnosis was performed predominantly in cases with more-severe disease. For an asymptomatic patient assessed following recovery from PCR-confirmed SARS-CoV-2 infection in this study, cutoff index values remained close to the positive/negative threshold for anti-SARS-CoV-2 antibodies. Therefore, the validity of our findings in ambulatory settings or for patients with asymptomatic/mildly symptomatic SARS-CoV-2 infection has yet to be shown and requires further study.

### Conclusion.

The Elecsys Anti-SARS-CoV-2 immunoassay demonstrated a very high specificity of 99.80% and a sensitivity of 99.5% for past infection in patients at ≥14 days after PCR confirmation, supporting its use as a potential tool for the identification of past exposure to SARS-CoV-2 infection. High assay specificity and sensitivity are crucial to ensure a high PPV for population screening, particularly in settings with low disease prevalence.

## References

[1] Li Q, Guan X, Wu P, Wang X, Zhou L, Tong Y, Ren R, Leung KSM, Lau EHY, Wong JY, Xing X, Xiang N, Wu Y, Li C, Chen Q, Li D, Liu T, Zhao J, Liu M, Tu W, Chen C, Jin L, Yang R, Wang Q, Zhou S, Wang R, Liu H, Luo Y, Liu Y, Shao G, Li H, Tao Z, Yang Y, Deng Z, Liu B, Ma Z, Zhang Y, Shi G, Lam TTY, Wu JT, Gao GF, Cowling BJ, Yang B, Leung GM, Feng Z 2020 Early transmission dynamics in Wuhan, China, of novel coronavirus-infected pneumonia. N Engl J Med 382:1199–1207. doi:10.1056/NEJMoa2001316. 31995857 PMC7121484

[2] World Health Organization. 2020 Pneumonia of unknown cause—China. Disease outbreak news, 5 January 2020. https://www.who.int/csr/don/05-january-2020-pneumonia-of-unkown-cause-china/en/.

[3] Fauci AS, Lane HC, Redfield RR 2020 Covid-19—navigating the uncharted. N Engl J Med 382:1268–1269. doi:10.1056/NEJMe2002387. 32109011 PMC7121221

[4] Lin L, Lu L, Cao W, Li T 2020 Hypothesis for potential pathogenesis of SARS-CoV-2 infection—a review of immune changes in patients with viral pneumonia. Emerg Microbes Infect 9:727–732. doi:10.1080/22221751.2020.1746199. 32196410 PMC7170333

[5] Loeffelholz MJ, Tang YW 2020 Laboratory diagnosis of emerging human coronavirus infections—the state of the art. Emerg Microbes Infect 9:747–756. doi:10.1080/22221751.2020.1745095. 32196430 PMC7172701

[6] Hamid S, Mir MY, Rohela GK 2020 Novel coronavirus disease (COVID-19): a pandemic (epidemiology, pathogenesis and potential therapeutics). New Microbes New Infect 35:100679. doi:10.1016/j.nmni.2020.100679. 32322401 PMC7171518

[7] Ahmed SF, Quadeer AA, McKay MR 2020 Preliminary identification of potential vaccine targets for the COVID-19 coronavirus (SARS-CoV-2) based on SARS-CoV immunological studies. Viruses 12:254. doi:10.3390/v12030254. PMC7150947 32106567

[8] Rokni M, Ghasemi V, Tavakoli Z 2020 Immune responses and pathogenesis of SARS-CoV-2 during an outbreak in Iran: comparison with SARS and MERS. Rev Med Virol 30:e2107. doi:10.1002/rmv.2107. 32267987 PMC7235481

[9] Li G, Fan Y, Lai Y, Han T, Li Z, Zhou P, Pan P, Wang W, Hu D, Liu X, Zhang Q, Wu J 2020 Coronavirus infections and immune responses. J Med Virol 92:424–432. doi:10.1002/jmv.25685. 31981224 PMC7166547

[10] Chan JFW, Yuan S, Kok KH, To KKW, Chu H, Yang J, Xing F, Liu J, Yip CCY, Poon RWS, Tsoi HW, Lo SKF, Chan KH, Poon VKM, Chan WM, Ip JD, Cai JP, Cheng VCC, Chen H, Hui CKM, Yuen KY 2020 A familial cluster of pneumonia associated with the 2019 novel coronavirus indicating person-to-person transmission: a study of a family cluster. Lancet 395:514–523. doi:10.1016/S0140-6736(20)30154-9. 31986261 PMC7159286

[11] Kampf G, Todt D, Pfaender S, Steinmann E 2020 Persistence of coronaviruses on inanimate surfaces and their inactivation with biocidal agents. J Hosp Infect 104:246–251. doi:10.1016/j.jhin.2020.01.022. 32035997 PMC7132493

[12] World Health Organization. 2020 Modes of transmission of virus causing COVID-19: implications for IPC precaution recommendations. Scientific brief, 29 March 2020. https://www.who.int/news-room/commentaries/detail/modes-of-transmission-of-virus-causing-covid-19-implications-for-ipc-precaution-recommendations.

[13] Wang D, Hu B, Hu C, Zhu F, Liu X, Zhang J, Wang B, Xiang H, Cheng Z, Xiong Y, Zhao Y, Li Y, Wang X, Peng Z 2020 Clinical characteristics of 138 hospitalized patients with 2019 novel coronavirus-infected pneumonia in Wuhan, China. JAMA 323:1061–1069. doi:10.1001/jama.2020.1585. 32031570 PMC7042881

[14] Huang C, Wang Y, Li X, Ren L, Zhao J, Hu Y, Zhang L, Fan G, Xu J, Gu X, Cheng Z, Yu T, Xia J, Wei Y, Wu W, Xie X, Yin W, Li H, Liu M, Xiao Y, Gao H, Guo L, Xie J, Wang G, Jiang R, Gao Z, Jin Q, Wang J, Cao B 2020 Clinical features of patients infected with 2019 novel coronavirus in Wuhan, China. Lancet 395:497–506. doi:10.1016/S0140-6736(20)30183-5. 31986264 PMC7159299

[15] Amanat F, Stadlbauer D, Strohmeier S, Nguyen THO, Chromikova V, McMahon M, Jiang K, Arunkumar GA, Jurczyszak D, Polanco J, Bermudez-Gonzalez M, Kleiner G, Aydillo T, Miorin L, Fierer DS, Lugo LA, Kojic EM, Stoever J, Liu STH, Cunningham-Rundles C, Felgner PL, Moran T, Garcia-Sastre A, Caplivski D, Cheng AC, Kedzierska K, Vapalahti O, Hepojoki JM, Simon V, Krammer F 2020 A serological assay to detect SARS-CoV-2 seroconversion in humans. Nat Med 26:1033–1036. doi:10.1038/s41591-020-0913-5. 32398876 PMC8183627

[16] Farnsworth CW, Anderson NW 2020 SARS-CoV-2 serology: much hype, little data. Clin Chem 66:875–877. doi:10.1093/clinchem/hvaa107. 32343775 PMC7197624

[17] Okba NMA, Müller MA, Li W, Wang C, GeurtsvanKessel CH, Corman VM, Lamers MM, Sikkema RS, de Bruin E, Chandler FD, Yazdanpanah Y, Le Hingrat Q, Descamps D, Houhou-Fidouh N, Reusken CBEM, Bosch BJ, Drosten C, Koopmans MPG, Haagmans BL 2020 Severe acute respiratory syndrome coronavirus 2-specific antibody responses in coronavirus disease 2019 patients. Emerg Infect Dis 26:1478–1488. doi:10.3201/eid2607.200841. 32267220 PMC7323511

[18] Xiao SY, Wu Y, Liu H 2020 Evolving status of the 2019 novel coronavirus infection: proposal of conventional serologic assays for disease diagnosis and infection monitoring. J Med Virol 92:464–467. doi:10.1002/jmv.25702. 32031264 PMC7167054

[19] Ni L, Ye F, Cheng ML, Feng Y, Deng YQ, Zhao H, Wei P, Ge J, Gou M, Li X, Sun L, Cao T, Wang P, Zhou C, Zhang R, Liang P, Guo H, Wang X, Qin CF, Chen F, Dong C 2020 Detection of SARS-CoV-2-specific humoral and cellular immunity in COVID-19 convalescent individuals. Immunity 52:971–977. doi:10.1016/j.immuni.2020.04.023. 32413330 PMC7196424

[20] Jiang S, Hillyer C, Du L 2020 Neutralising antibodies against SARS-CoV-2 and other human coronaviruses. Trends Immunol 41:355–359. doi:10.1016/j.it.2020.03.007. 32249063 PMC7129017

[21] Klasse PJ 2016 How to assess the binding strength of antibodies elicited by vaccination against HIV and other viruses. Expert Rev Vaccines 15:295–311. doi:10.1586/14760584.2016.1128831. 26641943 PMC4766047

[22] Iwasaki A, Yang Y 2020 The potential danger of suboptimal antibody responses in COVID-19. Nat Rev Immunol 20:339–341. doi:10.1038/s41577-020-0321-6. 32317716 PMC7187142

[23] Müller L, Ostermann PN, Walker A, Wienemann T, Mertens A, Adams O, Andree M, Hauka S, Lübke N, Keitel V, Drexler I, Di Cristanziano V, Hermsen DF, Kaiser R, Boege F, Klein F, Schaal H, Timm J, Senff T 2020 Sensitivity of commercial anti-SARS-CoV-2 serological assays in a high-prevalence setting. medRxiv https://www.medrxiv.org/content/10.1101/2020.06.11.20128686v2.full.pdf. 10.1007/s10096-021-04169-7 PMC7856849 33534090

[24] Luchsinger LL, Ransegnola B, Jin D, Muecksch F, Weisblum Y, Bao W, Jovvian George P, Rodriguez M, Tricoche N, Schmidt F, Gao C, Jawahar S, Pal M, Schnall E, Zhang H, Strauss D, Yazdanbakhsh K, Hillyer CD, Bieniasz PD, Hatziioannou T 2020 Serological analysis of New York City COVID19 convalescent plasma donors. medRxiv https://www.medrxiv.org/content/10.1101/2020.06.08.20124792v1.full.pdf.

[25] Chen Y, Li L 2020 SARS-CoV-2: virus dynamics and host response. Lancet Infect Dis 20:515–516. doi:10.1016/S1473-3099(20)30235-8. 32213336 PMC7156233

[26] Liu W, Liu L, Kou G, Zheng Y, Ding Y, Ni W, Wang Q, Tan L, Wu W, Tang S, Xiong X, Zheng S 2020 Evaluation of nucleocapsid and spike protein-based enzyme-linked immunosorbent assays for detecting antibodies against SARS-CoV-2. J Clin Microbiol 58:e00461-20. doi:10.1128/JCM.00461-20. 32229605 PMC7269413

[27] Guo L, Ren L, Yang S, Xiao M, Chang D, Yang F, Dela Cruz CS, Wamg Y, Wu C, Xiao Y, Zhang L, Han L, Dang S, Xu Y, Yang Q, Xu S, Zhu H, Xu Y, Jin Q, Sharma L, Wang L, Wang J 2020 Profiling early humoral response to diagnose novel coronavirus disease (COVID-19). Clin Infect Dis 71:778–785. doi:10.1093/cid/ciaa310. 32198501 PMC7184472

[28] To KK, Tsang OT, Leung WS, Tam AR, Wu TC, Lung DC, Yip CC, Cai JP, Chan JM, Chik TS, Lau DP, Choi CY, Chen LL, Chan WM, Chan KH, Ip JD, Ng AC, Poon RW, Luo CT, Cheng VC, Chan JF, Hung IF, Chen Z, Chen H, Yuen KY 2020 Temporal profiles of viral load in posterior oropharyngeal saliva samples and serum antibody responses during infection by SARS-CoV-2: an observational cohort study. Lancet Infect Dis 20:565–574. doi:10.1016/S1473-3099(20)30196-1. 32213337 PMC7158907

[29] Long QX, Liu BZ, Deng HJ, Wu GC, Deng K, Chen YK, Liao P, Qiu JF, Lin Y, Cai XF, Wang DQ, Hu Y, Ren JH, Tang N, Xu YY, Yu LH, Mo Z, Gong F, Zhang XL, Tian WG, Hu L, Zhang XX, Xiang JL, Du HX, Liu HW, Lang CH, Luo XH, Wu SB, Cui XP, Zhou Z, Zhu MM, Wang J, Xue CJ, Li XF, Wang L, Li ZJ, Wang K, Niu CC, Yang QJ, Tang XJ, Zhang Y, Liu XM, Li JJ, Zhang DC, Zhang F, Liu P, Yuan J, Li Q, Hu JL, Chen J, Huang AL 2020 Antibody responses to SARS-CoV-2 in patients with COVID-19. Nat Med 26:845–848. doi:10.1038/s41591-020-0897-1. 32350462

[30] Lou B, Li TD, Zheng SF, Su YY, Li ZY, Liu W, Yu F, Ge SX, Zou QD, Yuan Q, Lin S, Hong CM, Yao XY, Zhang XJ, Wu DH, Zhou GL, Hou WH, Li TT, Zhang YL, Zhang SY, Fan J, Zhang J, Xia NS, Chen Y 2020 Serology characteristics of SARS-CoV-2 infection since exposure and post symptom onset. Eur Respir J 2020:2000763. doi:10.1183/13993003.00763-2020. PMC7401320 32430429

[31] Zhao J, Yuan Q, Wang H 2020 Antibody responses to SARS-CoV-2 in patients of novel coronavirus disease 2019. Clin Infect Dis doi:10.1093/cid/ciaa344. PMC7184337 32221519

[32] Xiao AT, Gao C, Zhang S 2020 Profile of specific antibodies to SARS-CoV-2: the first report. J Infect 81:147–178. doi:10.1016/j.jinf.2020.03.012. PMC7118534 32209385

[33] Meyer B, Torriani G, Yerly S, Mazza L, Calame A, Arm-Vernez I, Zimmer G, Agoritsas T, Stirnemann J, Spechbach H, Guessous I, Stringhini S, Pugin J, RouxLombard P, Fontao L, Siegrist CA, Eckerle I, Vuilleumier N, Kaiser L, for the Geneva Center for Emerging Viral Diseases. 2020 Validation of a commercially available SARS-CoV-2 serological immunoassay. medRxiv https://www.medrxiv.org/content/10.1101/2020.05.02.20080879v1.full.pdf. 10.1016/j.cmi.2020.06.024 PMC7320699 32603801

[34] Li J, Fine J 2004 On sample size for sensitivity and specificity in prospective diagnostic accuracy studies. Stat Med 23:2537–2550. doi:10.1002/sim.1836. 15287083

[35] Tang MS, Hock KG, Logsdon NM, Hayes JE, Gronowski AM, Anderson NW, Farnsworth CW 2020 Clinical performance of two SARS-CoV-2 serologic assays. Clin Chem 66:1055–1062. doi:10.1093/clinchem/hvaa120. 32402061 PMC7239232

[36] Bryan A, Pepper G, Wener MH, Fink SL, Morishima C, Chaudhary A, Jerome KR, Mathias PC, Greninger AL 2020 Performance characteristics of the Abbott Architect SARS-CoV-2 IgG assay and seroprevalence in Boise, Idaho. J Clin Microbiol 58:e00941-20. doi:10.1128/JCM.00941-20. 32381641 PMC7383515

[37] Perkmann T, Perkmann-Nagele N, Breyer MK, Breyer-Kohansal R, Burghuber OC, Hartl S, Aletaha D, Sieghart D, Quehenberger P, Marculescu R, Mucher P, Strassi R, Wagner OF, Binder CJ, Haslacher H 2020 Side by side comparison of three fully automated SARS-CoV-2 antibody assays with a focus on specificity. medRxiv https://www.medrxiv.org/content/10.1101/2020.06.04.20117911v3. 10.1093/clinchem/hvaa198 PMC7454460 32777031

[38] Kohmer N, Westhaus S, Rühl C, Ciesek S, Rabenau HF 2020 Brief clinical evaluation of six high-throughput SARS-CoV-2 IgG antibody assays. J Clin Virol 129:104480. doi:10.1016/j.jcv.2020.104480. 32505777 PMC7263247

[39] Shen C, Wang Z, Zhao F, Yang Y, Li J, Yuan J, Wang F, Li D, Yang M, Xing L, Wei J, Xiao H, Yang Y, Qu J, Qing L, Chen L, Xu Z, Peng L, Li Y, Zheng H, Chen F, Huang K, Jiang Y, Liu D, Zhang Z, Liu Y, Liu L 2020 Treatment of 5 critically ill patients with COVID-19 with convalescent plasma. JAMA 323:1582–1589. doi:10.1001/jama.2020.4783. 32219428 PMC7101507

[40] Baruah V, Bose S 2020 Immunoinformatics-aided identification of T cell and B cell epitopes in the surface glycoprotein of 2019-nCoV. J Med Virol 92:495–500. doi:10.1002/jmv.25698. 32022276 PMC7166505

[41] Haveri A, Smura T, Kuivanen S, Österlund P, Hepojoki J, Ikonen N, Pitkäpaasi M, Blomqvist S, Rönkkö E, Kantele A, Strandin T, Kallio-Kokko H, Mannonen L, Lappalainen M, Broas M, Jiang M, Siira L, Salminen M, Puumalainen R, Sane J, Melin M, Vapalahti O, Savolainen-Kopra C 2020 Serological and molecular findings during SARS-CoV-2 infection: the first case study in Finland, January to February 2020. Euro Surveill 25:2000266 https://www.eurosurveillance.org/content/10.2807/1560-7917.ES.2020.25.11.2000266. 10.2807/1560-7917.ES.2020.25.11.2000266 PMC7096774 32209163

[42] Hou H, Wang T, Zhang B, Luo Y, Mao L, Wang F, Wu S, Sun Z 2020 Detection of IgM and IgG antibodies in patients with coronavirus disease 2019. Clin Transl Immunol 9:e01136. doi:10.1002/cti2.1136. PMC7202656 32382418

[43] Flahault A, Cadilhac M, Thomas G 2005 Sample size calculation should be performed for design accuracy in diagnostic test studies. J Clin Epidemiol 58:859–862. doi:10.1016/j.jclinepi.2004.12.009. 16018921

